# Supplementary material for: Grasping extreme aerodynamics on a low-dimensional manifold
Source: Nat Commun. 2023 Oct 14;14:6480. doi: 10.1038/s41467-023-42213-6 (PMC10576750; doi:10.1038/s41467-023-42213-6)
Supplement: Supplementary file 1 — Supplementary Information [file 41467_2023_42213_MOESM1_ESM.pdf]

Supplementary Information

Grasping Extreme Aerodynamics on a Low-Dimensional Manifold

Kai Fukami and Kunihiko Taira*
Department of Mechanical and Aerospace Engineering, University of California, Los Angeles, CA, USA
ktaira@seas.ucla.edu

Autoencoder Setup

We use an autoencoder composed of convolutional neural networks (CNN) [36] and multi-layer perceptrons (MLP) [37], as illustrated in Fig. 3 in the main text. Full details on the parameters of the present convolutional nonlinear autoencoder are shown in table S.1.

Reconstruction of vorticity fields

Comparative assessment of the reconstructed flow fields using different techniques is offered in this section. Here, we compare the reconstructed vorticity field from PCA, a regular autoencoder, and the lift-augmented autoencoder. To quantitatively assess the reconstruction performance, we evaluate the accuracy of the structural similarity index (SSIM) [41] between the reference and the decoded flow fields. The SSIM χ is defined as

$$\chi' = \frac{(2\mu_r\mu_d + C_1)(2\sigma_{rd} + C_2)}{(\mu_r^2 + \mu_d^2 + C_1)(\sigma_r^2 + \sigma_d^2 + C_2)}. \tag{1}$$

Here, subscripts r and d are used for the reference and decoded flow fields, μ and σ are the mean and standard deviation of given data, respectively, σ_{rd} is the covariance of the reference and decoded flow fields, and the constants $(C_1, C_2) = (0.16, 1.44)$ are used to stabilize division [41]. The SSIM χ lies between 0, representing no similarity, and 1, representing an identical image. The value of the SSIM is provided on every reconstructed vorticity field plot of the main text.

In this supplemental material, we present two cases of $(\alpha, G, L, y_0/c) = (40^\circ, -2.2, 0.5, 0.3)$ and $(60^\circ, -2.8, 1.5, 0)$ in Figs. S.1(a) and (b), respectively. We compare the performance of PCA, regular autoencoder, and the lift-augmented autoencoder all with the number of the latent vector being set to 3. As evident from these figures, PCA struggles to reconstruct high-dimensional states over time for both cases of extreme aerodynamic flows. PCA completely fails to detect the incoming gust vortex. In addition to the failure of reconstruction for the presence of the vortex disturbance upstream of the wing and the wake structures, limitations of linear reconstruction techniques are also observed around the wing with superpositions of wings appearing incorrectly.

In contrast, the reconstruction can be greatly improved with a nonlinear convolutional autoencoder, as shown in Fig. S.1. The position of extreme vortex disturbance is estimated very accurately over time including when a vortex gust impinges the wing causing strong nonlinear interactions among the wing, gust, and wake. This observation shows that the collection of the present extreme aerodynamic flow fields can be significantly compressed into only three variables using a nonlinear autoencoder. The improvement of the reconstruction by the nonlinear model can also be observed with an example of disturbed flow fields with two extreme gust vortices.

The impingement with these multiple gust vortices causes challenges to stable flight with extreme levels of nonlinear lift responses than single-gust cases. These unseen situations cannot be generally analyzed with conventional linear analysis. The reconstructed flow fields with two different types of

Table S.1: The network structure of the present lift-augmented convolutional autoencoder. The size of the convolutional filter L_c and the number of the filter K are shown for each convolutional layer as (L_c, L_c, K). The maxpooling/upsampling ratio M is shown for each maxpooling/upsampling layer as (M, M).

**Encoder**		**Decoder**		**Lift decoder**	
**Layer**	**Data size**	**Layer**	**Data size**	**Layer**	**Data size**
Input	(240, 120, 1)	Fully connected	(16)	Fully connected	(32)
Conv2D (3, 3, 32)	(240, 120, 32)	Fully connected	(32)	Fully connected	(64)
Conv2D (3, 3, 32)	(240, 120, 32)	Fully connected	(64)	Fully connected	(32)
MaxPooling2D (2, 2)	(120, 60, 32)	Fully connected	(256)	Output 2 (Lift coefficient)	(1)
Conv2D (3, 3, 16)	(120, 60, 16)	Fully connected	(288)		
Conv2D (3, 3, 16)	(120, 60, 16)	Reshape	(12, 6, 4)		
MaxPooling2D (2, 2)	(60, 30, 8)	Conv2D (3, 3, 4)	(12, 6, 4)		
Conv2D (3, 3, 8)	(60, 30, 8)	Conv2D (3, 3, 4)	(12, 6, 4)		
Conv2D (3, 3, 8)	(60, 30, 8)	UpSampling2D (5, 5)	(60, 30, 4)		
MaxPooling2D (5, 5)	(12, 6, 8)	Conv2D (3, 3, 8)	(60, 30, 8)		
Conv2D (3, 3, 4)	(12, 6, 4)	Conv2D (3, 3, 8)	(60, 30, 8)		
Conv2D (3, 3, 4)	(12, 6, 4)	UpSampling2D (2, 2)	(120, 160, 8)		
Reshape	(288)	Conv2D (3, 3, 16)	(120, 160, 16)		
Fully connected	(256)	Conv2D (3, 3, 16)	(120, 160, 16)		
Fully connected	(64)	UpSampling2D (2, 2)	(240, 120, 16)		
Fully connected	(32)	Conv2D (3, 3, 32)	(240, 120, 32)		
Fully connected	(16)	Conv2D (3, 3, 32)	(240, 120, 32)		
Fully connected (Latent vector)	(3)	Output 1 (Vorticity field)	(240, 120, 1)		

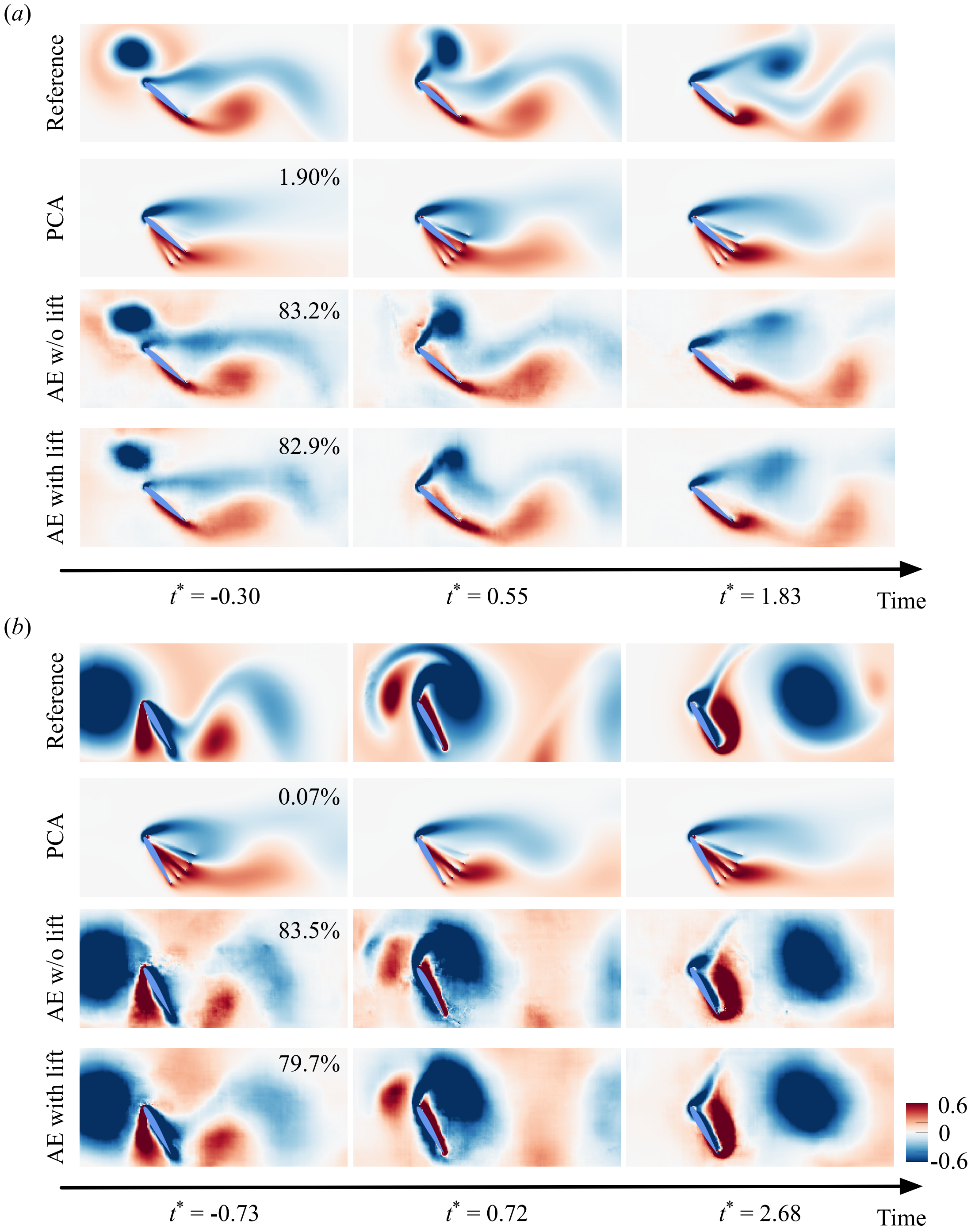

Figure S.1: Reconstructed vorticity fields by PCA, a regular autoencoder, and the present lift-augmented autoencoder for the cases of (a) $(\alpha, G, L, y_0/c) = (40^\circ, -2.2, 0.5, 0.3)$ and (b) $(60^\circ, -2.8, 1.5, 0)$. The values listed on the decoded flow fields are the time-averaged structural similarity indices between the decoded and reference fields.

vortex gust placements, (1) vertical and (2) horizontal arrangements, using PCA and the lift-augmented autoencoder are shown in Fig. S.2. As expected, PCA cannot detect the presence of two vortices and also completely fails to capture the influence of extreme disturbance on the wake over time. On the other hand, the present lift-augmented autoencoder is able to reconstruct the flow states, achieving over 90% SSIM for both examples. The ability to reconstruct such unseen complex gust situations based on a variety of single-vortex disturbed flow training data suggests that the discovered manifold space is indeed capturing the underlying extreme aerodynamic responses in not only a low-dimensional manner but also in a universal fashion. It is hence anticipated that the present manifold expression can be extended to wings flying into a wide range of extreme vortex-dominated gusts.

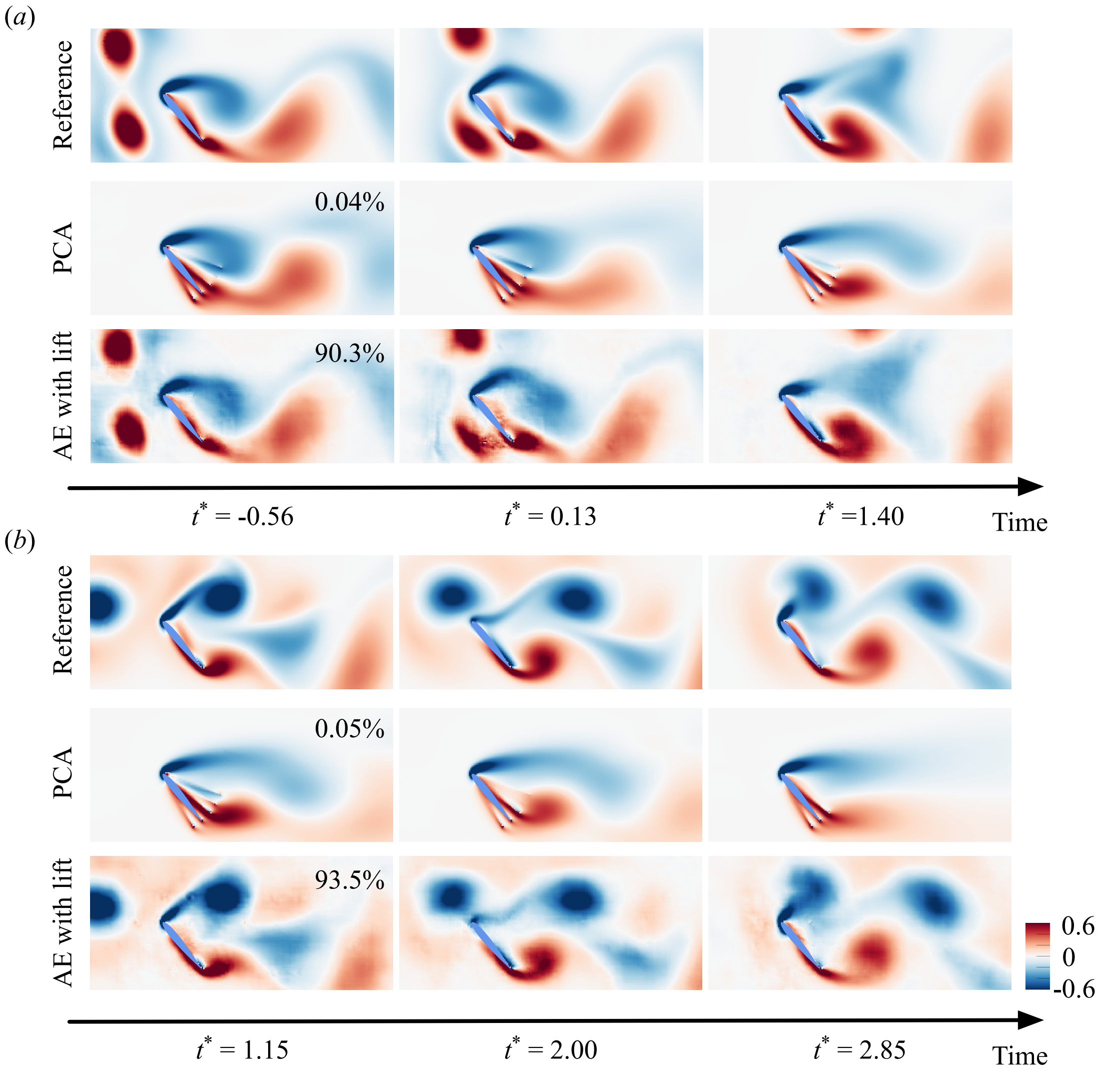

Figure S.2: Reconstructed vorticity fields by PCA and the present lift-augmented autoencoder for the cases of two gust vortices that are (a) vertically arranged and (b) horizontally arranged. The values listed on the decoded flow fields are the time-averaged structural similarity indices between the decoded and reference fields.
